# Optical Modeling of Plasmonic Nanoparticles with Electronically Depleted Layers

**DOI:** 10.1021/acs.jpcc.2c05582

**Published:** 2023-01-11

**Authors:** Nicolò Petrini, Michele Ghini, Nicola Curreli, Ilka Kriegel

**Affiliations:** † Functional Nanosystems, Istituto Italiano di Tecnologia (IIT), via Morego 30, 16163Genova, Italy; ‡ Dipartimento di Fisica, Università degli Studi di Genova, Via Dodecaneso 33, 16146, Genova, Italy

## Abstract

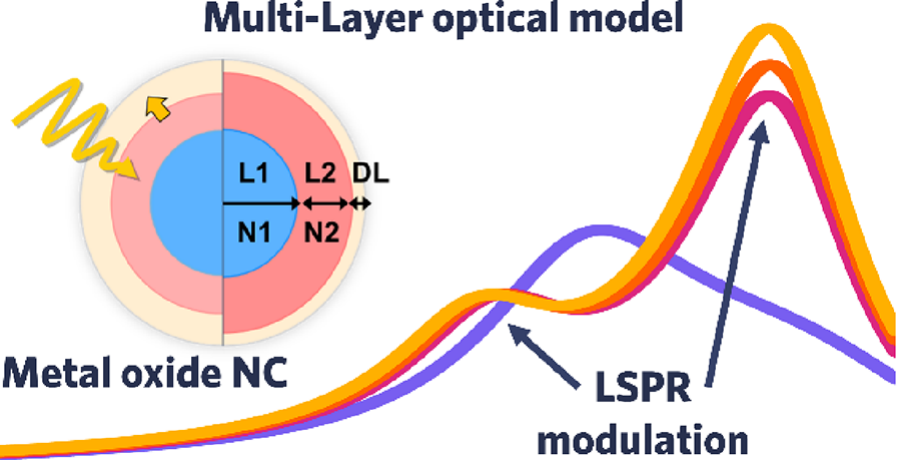

Doped metal oxide (MO) nanocrystals (NCs) are well-known for the localized surface plasmon resonance in the infrared range generated by free electrons in the conduction band of the material. Owing to the intimate connection between plasmonic features and the NC’s carrier density profile, proper modeling can unveil the underlying electronic structure. The carrier density profile in MO NCs is characterized by the presence of an electronically depleted layer as a result of the Fermi level pinning at the surface of the NC. Moreover, the carrier profile can be spatially engineered by tuning the dopant concentrations in core–shell architectures, generating a rich plethora of plasmonic features. In this work, we systematically studied the influence of the simulation parameters used for optical modeling of representative experimental absorption spectra by implementing multilayer models. We highlight in particular the importance of minimizing the fit parameters by support of experimental results and the importance of interparameter relationships. We show that, in all cases investigated, the depletion layer is fundamental to correctly describe the continuous spectra evolution. We foresee that this multilayer model can be used to design the optoelectronic properties of core–shell systems in the framework of energy band and depletion layer engineering.

## Introduction

Plasmonic nanocrystals (NCs) are being intensively investigated by the scientific community with the promise of a variety of applications, including surface-enhanced infrared spectroscopy over single molecule sensing, smart windows, and plasmon photothermal therapy.^1−6^ In particular, doped metal oxide (MO) NCs present a combination of several convenient features, such as electrical conductivity and optical transparency in the visible range, with strong and tunable localized surface plasmon resonances (LSPRs) in the near-infrared (NIR) region of the electromagnetic spectrum.^3,7,8^ Moreover, recent studies highlighted the possibility to store and subsequently release hundreds of extra carriers per MO NC.^9,10^ These carriers are accumulated after light absorption in a process called photodoping, opening interesting perspectives for light-driven energy storage and photocatalysis applications.^10−13^ For such applications, it is mandatory to extract fundamental electronic properties from the nanomaterial, which can be achieved either by direct electronic measurements through contact or indirectly by the analysis of the steady-state optical response. The latter is a powerful tool to noninvasively acquire useful optoelectronic information. In fact, the NC LSPRs are extremely sensitive to the morphological and electronic structure, such as the size and shape of the NC, the materials, doping levels, as well as the dopant placement at the nanoscale.^7,14−18^ In particular, a peculiarity of some doped MO NCs, such as Sn-doped In_2_O_3_ (ITO), is the upward bending of the conduction band close to the surface as a result of the presence of surface states and the consequent Fermi level pinning.^9,19,20^ This bending drastically reduces the carrier density in the superficial regions of the particle, resulting in a spatially varying carrier density profile. Part of the NC is entirely depleted of electrons and thus behaves as a dielectric. This region is typically referred to as the depletion layer (DL) and has extensions in the nanometer regime.^16,17,19,21^ Given the small sizes of the MO NCs (radii in the few to tens of nanometer regime) and their high surface-to-volume ratio, it becomes clear that such DL has a strong influence on their electronic and optical properties as well.

With the correct choice of models, it is possible to extract fundamental properties of the nanomaterials’ electronic structure, the free carrier concentration, and its spatial profile in the NC.^9^ In the past, several models have been used to describe the properties of highly doped MO NCs, ranging from finite element methods (numerical) to simplified models based on the effective medium approximation (analytical). In this latter approach, the carrier density profiles are approximated as step functions, and each region so defined is represented by a specific dielectric function. Their description ranges from core–shell systems (two-layer model) to multilayer structures, such as three-layer models and beyond, and has been implemented for various material systems.^9,16,19^ More recently, the interest for a complete description of the plasmonic response with multilayer models has risen as a tool to describe the dielectric properties of doped MO NCs and their superficial DL.^16,19,20^ With the increase of publications in this field and the development of synthesis protocols to control the dopant distribution and core–shell structures,^8,9,15^ it becomes more and more important to establish a common ground for applying such models to make the results comparable within the scientific community. For this reason, in this work, we outline a step-by-step guide on the use of multilayer models for plasmonic nanocrystals. Specifically, we systematically consider the optical parameters employed in the models and show the effects of their tuning in simulations, which aim at highlighting the influence of a specific parameter variation over the total absorption spectra. Differently from the previous reported works, we carefully consider the interdependence of the simulation parameters, in particular, the damping factor relation with carrier concentration and layer size, including such dependence in the simulation results. Then, we discuss the implementation of such optical models to describe experimental optical spectra evolution, discussing the importance of the minimum number of layers necessary to describe a system and emphasizing the importance of each parameter in light of the previous simulation results. We highlight the minimal use of fit parameters to avoid the possible overfitting and the fitting boundaries, which have to be chosen to give the physical consistency of the fitting results, which otherwise are subject to overfitting and artifacts. Specifically, we evaluate multilayer models to describe the optical response of ITO and ITO-In_2_O_3_ (ITO-IO) NCs, and we highlight the importance of taking into account the DL in both cases. We implement data extracted from the literature as case studies for the multilayer models. We display their effectiveness by describing three particular cases that are not readily explainable without multilayer models. Although we limit ourselves to the description of ITO-based nanostructures, we highlight that these models are valid also for other material systems where depletion layer formation plays a detrimental role.^9^ This manuscript is intended to be a tutorial to raise common standards on multilayer optical modeling and to make such methods accessible also to the nonexperts.

## Methods

Numerical simulations are capable to calculate precisely the band profile bending induced by Fermi level pinning by solving the Poisson equations.^17,19^ Nevertheless, this method is computationally expensive, and the picture of the carrier density profile can be simplified by implementing empirical models that approximate the NC geometry as concentric layers with associated dielectric function. The advantage of this approach is reducing the number of parameters, allowing for fitting the optical response and extracting the electronic features, and hence giving direct access to the physical parameters related to their optical response. The most common example is the simplification of the depletion layer as an undoped dielectric shell, which surrounds the doped core of the same material. Most models found in the literature rely on the Mie solution and the assumption of Drude free electrons to describe the absorption spectrum of doped MO NCs. The Maxwell–Garnett effective medium approximation (EMA) can refine this picture.^9,20,22,23^ It considers multiple inclusions of materials with specific dielectric properties within a surrounding dielectric medium.^12^ The EMA could in principle be employed to accurately describe the continuous modulation of the carrier density profile that results from the Fermi level pinning by using an iterative approach. In this case, infinitesimally thin layers are taken into account, each with specific dielectric properties.^19^ However, such approach requires the use of numerical tools as well and a precise in-depth knowledge of the underlying electronic structure of the system to be able to successfully employ the EMA model, albeit with high accuracy. Moreover, the large number of parameters associated to each layer can represent a source of overfitting and artifacts in fitting results.^19^ Several examples have already shown that the approximation of the system with a step function carrier density profile as the input to the optical model allows representing well the optical response of the material. For example, Agrawal et al. implemented a two-layer model to describe the optical response of uniformly doped Sn:In_2_O_3_ (ITO) by taking into account a constant carrier density in the core region and a depleted shell region (i.e., a dielectric).^20^ The central finding of this work was related to the expansion of the doped core radius at the expense of the (undoped) depletion layer upon electrochemical charging.^20^ Recently, the two-layer formula was applied by Gibbs et al. for core–shell ITO-IO NCs as well, where each layer of the model described a material with different properties.^22^ In a recent work of ours, we showed that the resulting nonuniform carrier density profile in core–shell ITO-IO NCs is well approximated with a minimum of three electronically distinct regions, i.e., the (heavily doped) core, a shell (with slightly lower carrier density), and the depletion layer (DL).A three-layer model was implemented.^9^ In the following chapter, we give a general introduction to the optical modeling and the EMA approach before describing in detail the examples of two-layer and three-layer models together with the role of fit parameters.

### General Remarks on Optical Modeling

The optical response of an NC that is illuminated by a beam of light can be physically modeled by considering the light scattered by the particle and the light that is absorbed. Scattering and absorption of a spherical NC depend specifically on three variables: the nature of the material composing the NC (i.e., dielectric function), the size (i.e., radius) of the NC, and the wavelength of the impinging light. The dielectric function is an important parameter defining the material optical properties and can be approximated by different physical models depending on the material under consideration, i.e., if a metal or a dielectric. Metallic materials’ dielectric function can be modeled in the semiclassical limit by the Lorentz–Drude model, whereas for dielectric materials, a real (lossless) dielectric function can be employed. The LSPR of plasmonic NCs typically arises in the metallic regime as a result of the collective oscillations of free electrons.

For our purpose, we classify the properties of the MO into metallic or dielectric by the range of carrier densities. We highlight here that for MO the interband Lorentz-like contribution can be typically ignored because the LSPR energetic region is far beyond the bound Lorentz oscillator region.^24−29^ Hence, for MO NCs’ metallic regime, we implement the Drude free-electron model.^22^ In general, the Mott criterion^30^ represents the theoretical threshold between metallic and dielectric behavior, but specific thresholds could be further refined depending on the materials, associated Bohr exciton radius, and size of the layers. Materials with sufficiently low carrier density, typically below the critical carrier density given by the above-mentioned Mott criterion, can be considered as purely dielectric in the region of interest and hence are represented with a constant dielectric function. In the case of ITO NCs, the Mott threshold is calculated to be 6.3 × 10^18^ cm^–3^ from the Bohr radius.^17^ However, the value of 10^20^ cm^–3^ is usually considered in the literature as the threshold at which the plasmon becomes experimentally detectable,^9,17^ and in this work, we adopt the latter criterion.

For NCs affected by depletion layers and NCs synthesized in core–shell architectures, the internal structure can be decomposed in several regions with related carrier density levels that can be dielectric or metallic. It is important to distinguish these layers and associate to each of them the respective dielectric function and model. Consequently, the effective dielectric function of the whole NC system can be calculated by implementing appropriate models, i.e., the EMA. This physical quantity contains information about the different materials involved and the volume they are occupying in the NC.^23^ Then, for modeling the optical response of a colloidal NC solution, the effective dielectric function of the single NC is used to calculate the solution extinction cross section with the Mie solution approach. This methodology allows calculating the extinction coefficient of small noninteracting spheres dispersed in a dielectric medium. Eventually, the extinction cross section is employed in the Lambert–Beer law to calculate the optical density of the NC dispersion. In the NC size range investigated (radius smaller than 15 nm), the scattering contribution to the optical response can be neglected, leading to the equivalence of extinction and absorption coefficients. This approximation can be verified by considering the analytical solution of Maxwell equations in the case of an electromagnetic field interacting with a spherical particle, as explained in the Supporting Information (Figure S1). For the sake of completeness, it must be highlighted that quantum confinement effects are negligible because the NC radii are typically larger than the Bohr exciton radius (in ITO from 1.3 nm^29^ to 2.38 nm^31^ to 5 nm^32^), and the quantization energy associated to these NC sizes is much lower than the energy associated to the plasma frequency.^9^ Nevertheless, these assumptions need to be evaluated individually depending on which material is studied.

### Optical Two-Layer and Three-Layer Models

**Figure 1:**
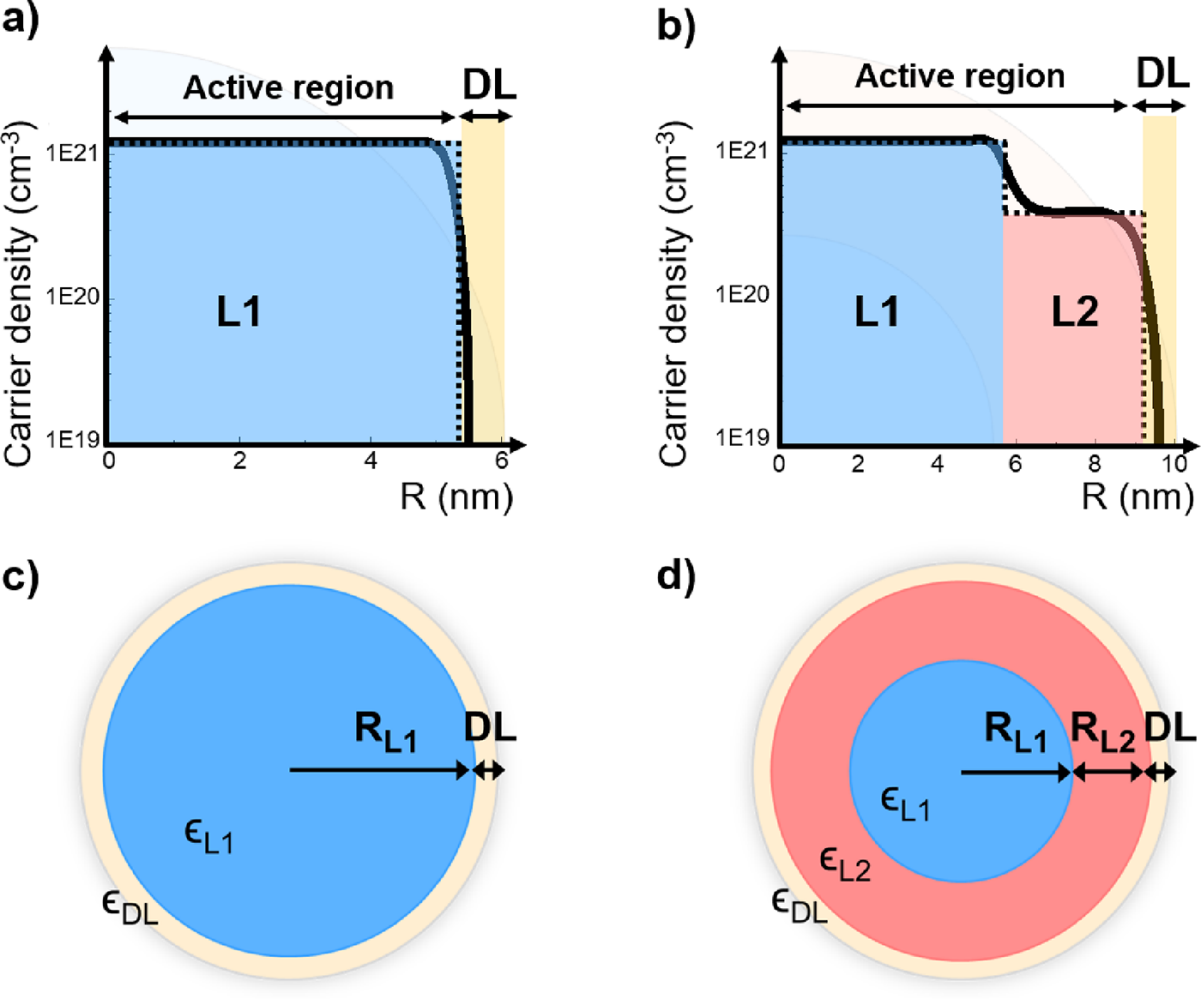
Multilayer optical models. Carrier density profiles for (a) homogeneous ITO and (b) core–shell ITO-In_2_O_3_ nanocrystals. The continuous black line is obtained by numerical simulations from ref (9), whereas the dotted line illustrates the step-function approximation implemented here. The active region is formed by layer 1 (in blue) and layer 2 (in red, where present), whereas the electronically depleted region (DL) is depicted in yellow. The corresponding multilayer optical models developed for (c) homogeneous ITO and (d) core–shell ITO-In_2_O_3_ nanocrystals are depicted. The first two layers (*L*_1_ and *L*_2_) are characterized by the dielectric constants ε_*L*1_(ω) and ε_*L*2_(ω), whereas the depletion layer (DL) at the NC’s surface is described as a dielectric with a fixed ε_DL_.

In the following, we will discuss the fundamental equations describing the absorption of doped metal oxide NC solutions. The presence of superficial depletion layers in homogeneously doped ITO and core–shell ITO-IO NCs justifies the need for multilayer optical models. Figure 1 depicts the case of a uniformly doped ITO NC (Figure 1a) and an ITO-IO core–shell structure (Figure 1b) with the respective continuous carrier density profile as calculated by numerical models (black continuous lines) taken from ref (9). In both cases, the Fermi level pinning at the surface, deriving from the surface states, induces a bending in the carrier density distribution, which determines the carrier density profile and the depletion layer width.^9^ The simplified picture assuming that the NC dielectric structure can be approximated by constant dielectric layers as a step-function-like profile is illustrated as black dotted lines (Figure 1a,b, respectively). Consulting the illustration, it becomes obvious that in the first case (Figure 1a,c), it is necessary to take into account at least a two-layer model, considering a highly doped core (*L*_1_) and an undoped region (DL, i.e., the depletion region). Instead, in core–shell NC (Figure 1b,d), the carrier density profile is better approximated by three regions, accounting, respectively, for the core (*L*_1_), the shell (*L*_2_), and the depletion layer (DL). With the same considerations, it is easy to extend this procedure to multiple-layer geometries.

As mentioned above, for each material that composes the nanostructure and occupies a layer *L_n_* with a sufficient carrier density to support a detectable plasmon resonance (defined here as *n_e_Ln__*> 10^20^ cm^–3^),^9,17^ we employ a Drude-like formulation to describe its dielectric function ε_*Ln*_(ω):
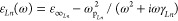
1where  is the bulk plasma frequency. Here, ε_∞_*Ln*__ is the bulk high-frequency dielectric permittivity, γ_*Ln*_ is the damping parameter, *e* is the electron charge, ε_0_ is the vacuum permittivity, and m* is the effective electron mass. Although all these values can be typically found in the literature, the bulk plasma frequency holds the key relationship with the carrier density *n_e_Ln__*, which strongly determines the dielectric function ε_*Ln*_. The damping parameter γ_*Ln*_ can be related to the carrier concentration *n_e_Ln__* and to the physical dimensions of the occupied region *R_Ln_* (considering a spherical geometry) as^33^
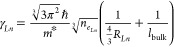
2where *ℏ* is the Plank’s constant and *l*_bulk_ is the electron mean free path of the bulk material.

Instead, for the electronically depleted layer (i.e., the DL), we use a constant dielectric function ε_DL_(ω) corresponding to the bulk high-frequency dielectric permittivity ε_∞_DL__:
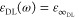
3This information can then be used to calculate the total effective dielectric function ε_eff_(ω) for an ensemble composed of two different materials *A* and *B*, employing the Maxwell–Garnett effective medium approximation mixing formula:
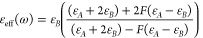
4where  is the volume ratio between the volume of layer *A* and the total volume (A + B).

Specifically, in the case of uniform doped NC, the total dielectric function is calculated by considering a two-layer model (2L-model) accounting for the core carrier density (*A* = *L*_1_ region) and for the depletion region (*B* = DL), as shown in Figure 1c. Hence, eq 4 becomes
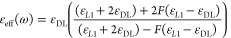
5where  accounts for the geometric volume ratio determined by the core and depletion region. *R*_*L*1_ and *R*_NC_ correspond to the radius of the doped core (*L*_1_) and the overall radius of the NC, respectively (Figure 1a,c).

Conversely, for the case of core–shell NCs, an additional shell layer is introduced; thus, a three-layer model (3L-model) is required. In formulas, eq 4 needs to be applied iteratively twice. In the first instance, the two layers *A* and *B* are respectively associated with the core (*A* = *L*_1_) and shell (*B* = *L*_2_) effective regions (see Figure 1d), which we denote altogether as the *active region*. This leads to obtaining an effective dielectric function for the active region, denoted as ε_active_(ω):
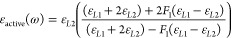
6where  accounts for the volume ratio of the core and shell in the active region, *V*_*L*1_ is the volume of the core region, *V*_*L*1 + *L*2_ is the volume of the active core (the core and shell region), *R*_*L*1_ is the core radius, and *R*_*L*1 + *L*2_ is the radius of the active region (Figure 1b).

In the second iteration instead, the active region corresponds to layer *A* with the just calculated dielectric function ε_active_(ω) and with an associated radius *R* = *R*_*L*1 + *L*2_; besides, layer *B* coincides with the depletion layer (DL). The final dielectric function of a single core-shell NC is then
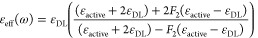
7where , with *V*_NC_ representing the total NC volume and *R*_NC_ the total NC radius.

Eventually, for all the described cases, the absorption can be calculated as the Mie solution in the quasi-static approximation, considering just the dipole mode^34^ and using the computed effective dielectric function of a single NC. Considering the scattering term as negligible, which is the case of the considered colloidal dispersed solution, the absorption ABS(ω) can be evaluated as
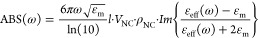
8where ε_m_ is the dielectric constant of the medium, ω is the angular frequency, *l* is the light path-length through the cuvette solution, *V*_NC_ is the volume of a single NC, and ρ_NC_ is the NC concentration in the solution.

It is evident that this approach can be extended in theory to an arbitrary number of layers, giving the possibility not only to describe NCs composed of many layers of different materials but also to approximate the continuous carrier density profile, as reported by Zandi et al.^19^

### Discussion of Parameters for Optical Models

To use the model described to simulate the absorption of nanoparticles or to fit experimental spectra, it is necessary to define all the parameters involved. In the following, we suggest a possible step-by-step procedure to determine or constrain each variable. We discuss the physical role of the fit parameters, and we analyze the optoelectronic information that can be extracted.

First, the optical path length (*l*) is a known value typically provided from the cuvette manufacturer, whereas the NC average radius (*R*_NC_) and the NC solution concentration (ρ_NC_) can be determined experimentally. Specifically, *R*_NC_ can be measured via electron microscopy (e.g., via TEM or SEM) by assuming spherical particles with a normal distribution of sizes, acquiring sufficient statistics with the help of an image acquisition software to convert circular areas into radii. From the average radius *R*_NC_, the NC total volume can be calculated. The concentration of the NC solution (ρ_NC_) can be obtained by means of inductively coupled plasma mass spectrometry (ICP-OES) measurements, combining the information on the NC volume, mass, and elemental composition. Once these measurements have been performed, *l*, *R*_NC_, and ρ_NC_ can be treated as fixed parameters in the simulations/fit, reducing the number of free parameters to evaluate in the multilayer model. Next, we proceed with the choice of the correct model, i.e., number of layers, for the material system analyzed. In the case of the uniformly doped ITO NC, it is sufficient to consider a two-layer model, as illustrated above, with *F*, *n*_*e*_*L*1__, and γ_*L*1_ being the simulation or fitting parameters. These parameters are strictly related to the quantities of *N*, *R*_*L*1_ and DL that serve to deliver a physical picture. In particular, *F* is given as the ratio between the volume occupied by the active region  and the total volume of the NC  by considering that *R*_*L*1_ ≤ *R*_NC_. As *V*_NC_ is a known value, from the fit of *F*, it is possible to obtain the active region radius *R*_*L*1_ and thus the complementary depleted region DL (as DL = *R*_NC_ – *R*_*L*1_). Moreover, typical values of DL are in the order of nanometers,^9,16,17,19^ further limiting the possible physically meaningful values of *R*_*L*1_. Instead, from the fit value *n*_*e*_*L*1__, the total number of carriers *N* contained in the active region *L*_1_ can be calculated as . An upper limit to the number of carriers *N* in the as-synthesized case can be obtained from the number of doping atoms present in the NC. A typical approach would be to do ICP-OES from a known amount of ITO NCs that have been digested in aqua regia [HCl/HNO3 3:1 (v/v)] overnight.^9^ The ICP-OES analysis will allow determining the ratio of Sn versus In and hence give an estimate of the doping concentration. Assuming that each Sn atom delivers one electron would give the upper limit to *N* the number of free carriers per NC. From this discussion, we can see that the fit parameter  contains information on the geometrical distribution of carriers inside the NC, whereas the carrier concentration  is related to the doping, i.e., to the number of free carriers in the material at any stage of the experiment. Eventually, the value of γ_*L*1_, which is the damping parameter, is material-specific, and its value for bulk case can be found in the literature.^3,22,28^ However, for NCs, this parameter is additionally related to the geometrical distribution, to the confinement of the carriers, and to the carrier concentration, being , recalling eq 2.^33^ Because this parameter depends on the bulk mean free path, which ranges from 5 to 17 nm for ITO and depends on the carrier concentration, the damping parameter cannot be fixed by evaluating eq 2. Instead, it must be considered as a fitting parameter free to vary, imposing the constraints and proportionalities dictated by the maximum and minimum bulk mean free path and by the evolution of layer thickness and carrier density concentration. Taking this discussion into account, we have now limited the amount of free parameters for the fit to two: *F*, related to *R*_*L*1_ with upper limit *R*_NC_, and *n*_*e*_*L*1__, related both to *R*_*L*1_ and to the number of carriers *N*, with the upper limit extracted experimentally. The value of γ_*L*1_ depends on the previous two and in this two-layer case will determine the width of the peak, and hence, it is recommended to give this parameter a certain freedom to adjust during the fit.

In the case of core–shell ITO-IO NCs, we recommend the three-layer model, which requires six parameters: *n*_*eL*1_, *n*_*eL*2_, *F*_1_, *F*_2_, γ_*L*1_, and γ_*L*2_. In fact, whenever a Drude-like material layer is added (*L_n_*), the set of parameters is enlarged by a new triplet of *n_e_n__*, *F_n_*, and γ_*n*_. In this type of fit, a level of complexity is added as now the geometrical carrier distribution is hidden in both *F*_1_ and *F*_2_. By knowing the total radius *R*_NC_, the respective portion of DL and *R*_active_ = *R*_*L*1_ + *R*_*L*2_ can be calculated from *F*_1_=  and *F*_2_= . The constraints here are more difficult to be defined: the maximum *R*_active_ is equal to *R*_NC_, but *R*_*L*1_ and *R*_*L*2_ depend on the electron rearrangement inside the NCs and can differ from the physical dopant distribution inside the NC. The total number of carriers in the NC is *N* = *N*_1_ + *N*_2_, which is related to the carrier density *n*_*eL*1_ and *n*_*eL*2_. The damping factors γ_*L*1_and γ_*L*2_ are respectively proportional to the carrier concentration *n*_*eL*1_ and *n*_*eL*2_ and present a common constant term, given by the bulk damping contribution, and a surface term, which is associated specifically to the layer size; hence,  and . Similar to the two-layer case, here, the damping factors γ_*L*1_and γ_*L*2_ must also be considered as fitting parameters because the bulk electron mean free path *l*_bulk_ does not have a fixed value. Because the total number of parameters increased compared to the two-layer case and fewer constraints can be determined, the three-layer case presents the problem of overfitting, and physically meaningful results must be carefully considered. In particular, there can be many combinations of the parameters describing layers *L*_1_ and *L*_2_ that can fit the optical spectra. To determine which one is physically more correct, it is usually necessary to consider a set of NCs with either different internal geometry (core/shell ratio) or different doping profiles.

Eventually, we remark here that, in the multilayer approach, a perfect distribution of sizes and doping levels in the NCs is usually assumed, which might not be accurate in all cases.^33,35^ In fact, as investigated by Gibbs et al.,^33^ the polydispersity of the NCs radii and heterogeneous doping can modify the total absorption spectra of the NC solution, and heterogeneous ensemble Drude approximation (HEDA) is necessary to correctly fit the optical spectra. However, the consequence for including such distribution is an increase in fitting parameters’ number, which can lead to overfitting issues, particularly when considering more than two layers. It is thus necessary to evaluate for each case if the fitting results assuming a perfect homogeneity are physically reasonable or if heterogeneity must be considered. In particular, the damping parameter and its evolution can be observed because the effect of polydispersity affects the LSPR broadening and can result in an apparent damping parameter for the homogeneous case, which is lower than the one obtained in the heterogeneous case. In the study cases analyzed in the following sections, we considered perfectly homogeneous solutions because a physical evolution of damping parameter was found considering the presence of depletion layers at the surface.

## Results and Discussion

In the following sections, we simulate the effect of tuning the aforementioned optical parameters to study the corresponding changes on the LSPR by accounting also for the interdependence between the parameters. We always consider in particular the dependence of the damping parameter on the corresponding electron density and layer size in both the two-layer and three-layer cases. Then, we implement these models to discuss three relevant cases recently published. We analyzed the experimental data reported in the literature (with corresponding citations and acknowledgements) to replicate the reported data with the multilayer model.^9,22^ In the first instance, we study the case of uniformly doped NCs’ spectral variation upon photodoping. We highlight the minimal use of parameter variation to describe the observed spectral evolution, where *N* (number of electrons) is increasing. We show that it is sufficient to change only *F* to describe the observed spectra changes upon photodoping. Thereafter, we implement the three-layer model to describe the shell growth in core–shell ITO-In_2_O_3_ NCs. The three-layer model has first been identified in a recent article published by the authors.^9^ The key novelty is to retain the initial total number of free electrons *N* constant upon evolution of the undoped shell and the detailed discussion on the determination of the maximum *N* upon fitting. Our fit results suggest that the constant number does not change, but carriers instead rearrange into a new spatially varying carrier density profile. In this case, the change of more parameters (*n*_*eL*1_, *n*_*eL*2_, *F*_1_, *F*_2_) is necessary to explain the observed spectral variation. Eventually, we discuss a peculiar case of core–shell NC absorption spectrum change upon photodoping, specifically plasmon splitting. These effects are not explainable with a simple two-layer model, highlighting the requirement of the third layer (DL) accounting for the carrier depletion.

### Parameter Simulations for Two-Layer and Three-Layer Models

**Figure 2:**
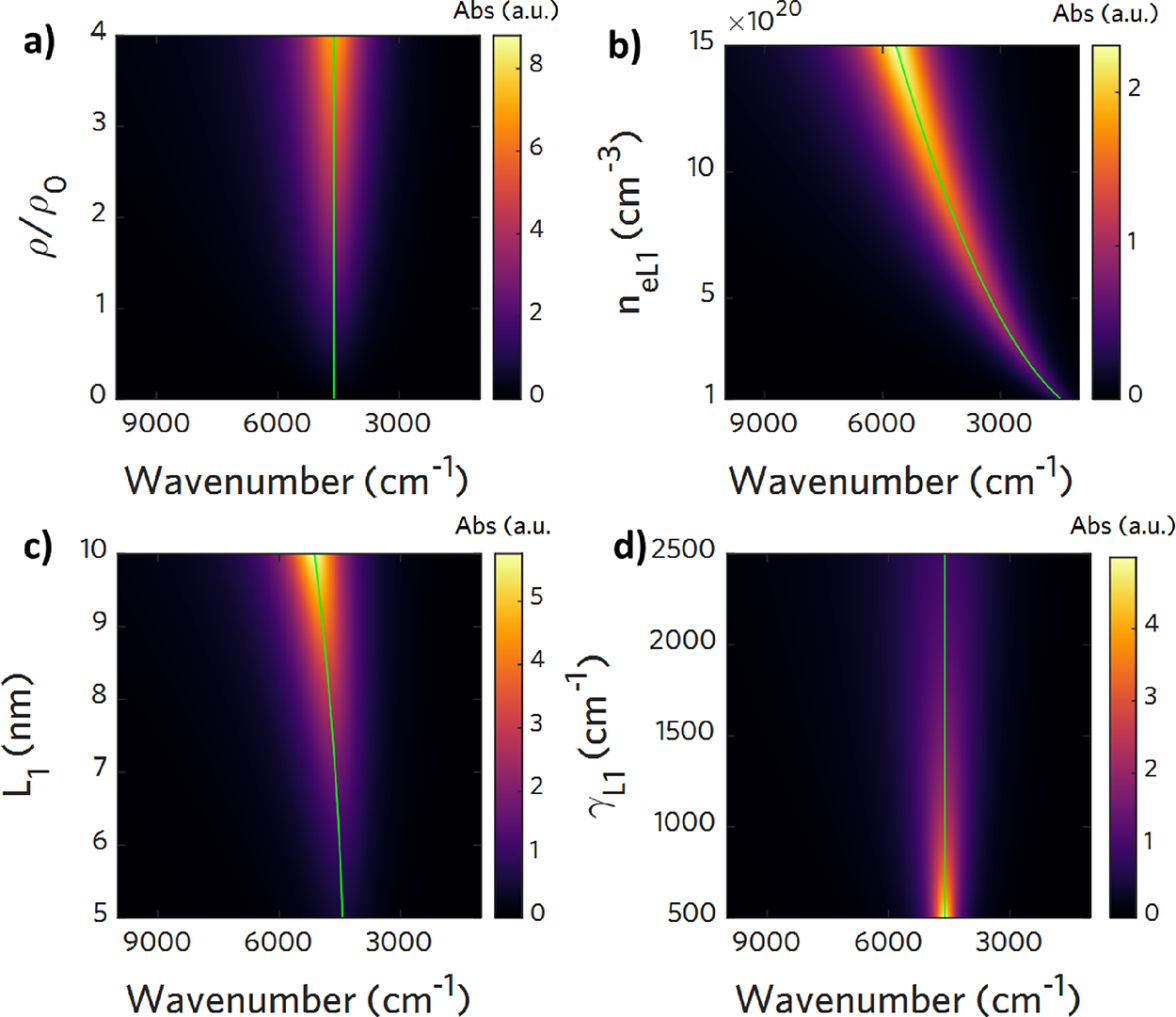
Spectral maps of simulated evolution of the absorption spectrum of homogeneous ITO NCs as a function of several parameters of the multilayer (two-layer) optical model. The map colorbar intensity is proportional to the LSPR intensity, and the peak position is highlighted with a green line. (a) Increasing the concentration of the NC solution ρ_NC_ with respect to a base concentration ρ_0_ = 6.7 · 10^12^ mL^–1^ increases linearly the absorption intensity maximum of the LSPR. An increase of (b) the carrier density level *n*_*e*_*L*1__ or (c) the volume associated with the core *L*_1_ significantly affects the peak lineshape, blueshifting the peak position and increasing its intensity. (d) Changes in the damping parameter affect both the width of the LSPR and its intensity but not the peak frequency position.

When considering the absorption spectrum, it is fundamental to discriminate the influence of each of the simulation or fitting parameters on the plasmonic peak position, intensity, and shape. To evaluate these changes, we simulated the optical spectrum changing selectively one parameter and reporting the obtained results in spectral maps with wavenumber in the abscissa and the parameter changes in the ordinate using colormaps to reproduce the intensity of the optical response. The default optical parameter values used for simulations in the two-layer case and three-layer case are reported, respectively, in Table S1 and S2 in the Supporting information. For the simpler two-layer case, recalling eq 8, it is evident that the peak intensity is directly proportional to the NC volume *V*_NC_ and to the solution concentration ρ_NC_. Therefore, an inaccurate estimation of these two parameters affects the maximum intensity of the plasmon peak. In Figure 2a, we report the simulation showing the spectra variation when the solution concentration changes relative to the base concentration ρ_0_. As we can see, the peak position is not affected by the relative change of concentration. On the contrary, as seen in Figure 2b,c, the carrier concentration *n*_*e*_*L*1__ and the inner layer dimension *L*_1_ (which is related to the volume ratio *F*) affect in the first place the peak position: increasing the electron concentration or increasing *L*_1_ blueshifts the peak while increasing its intensity as well. Both of these two simulations take into account not only the variation of *n*_*e*_*L*1__and *L*_1_ but also the change in γ_*L*1_ according to the proportionality previously reported. Eventually, assuming a single population of NCs with the same size, the resulting peak shape is determined only by the damping parameter γ_*L*1_: an increase in damping broadens the spectrum, whereas a decrease narrows it and enhances its intensity (Figure 2d). No peak shifts are observed.

**Figure 3:**
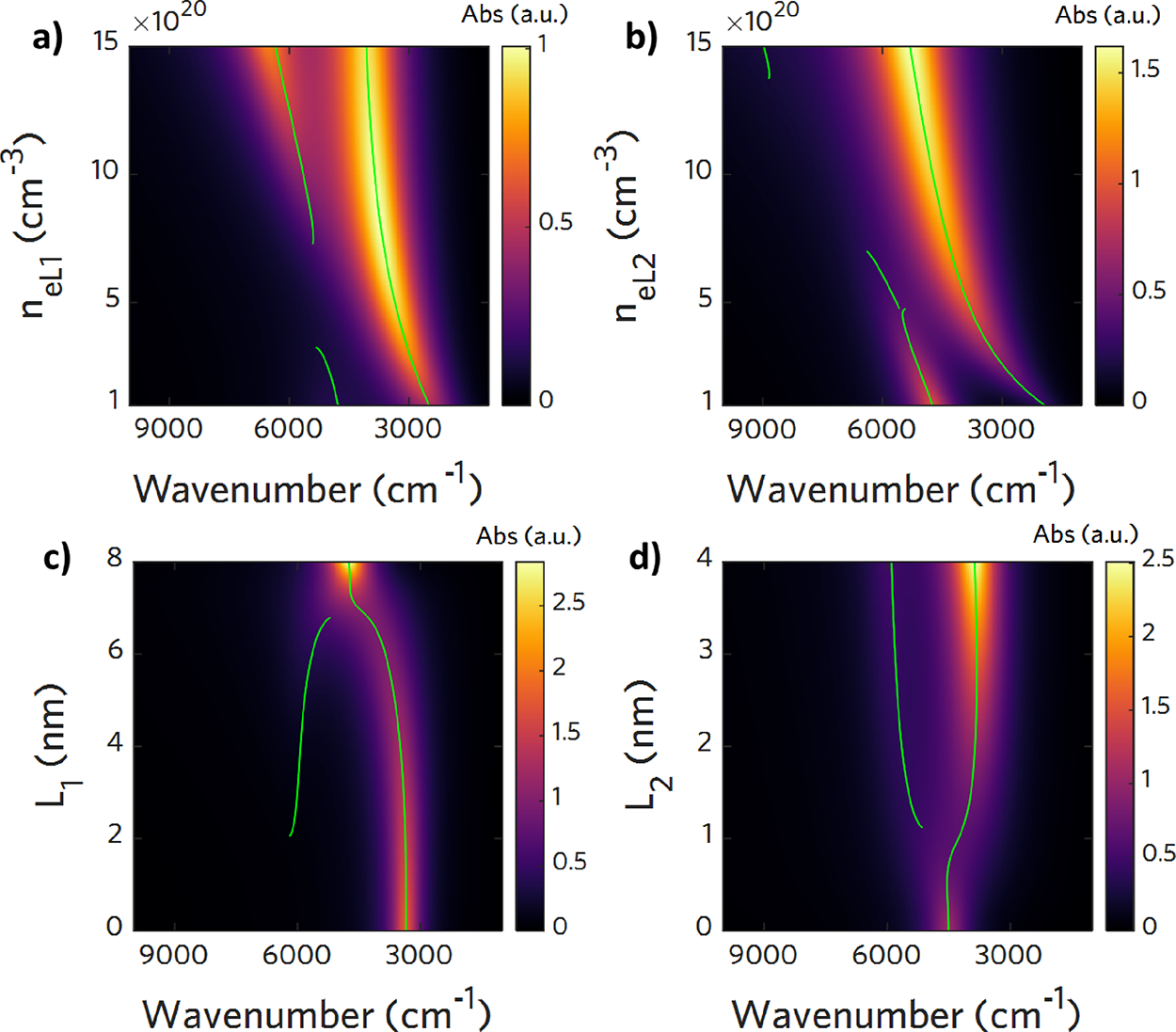
Spectral maps of the simulated evolution of the absorption spectrum of core/shell ITO/IO NCs as a function of several parameters of the multilayer (three-layer) optical model. The map colorbar intensity is proportional to the LSPR intensity, and the position of the main and shoulder peak (when present) is made visible by a greenline. (a, b) The effect of carrier density variation, respectively, *n*_*e*_*L*1__ and *n*_*e*_*L*2__, by keeping the inner layer structure constant. In both cases, we notice the nontrivial evolution shoulder peaks that disappear when *n*_*e*_*L*1__∼*n*_*e*_*L*2__ and the blueshift for both increasing *n*_*e*_*L*1__ and *n*_*e*_*L*2__. (c) The optical spectra evolution consisting of the main and double peaks merging when expanding the core region *L*_1_ to the detriment of the shell *L*_2_, leaving the depletion layer unaffected. (d) The effect of maintaining the core *L*_1_ fixed and expanding the shell *L*_2_ in the depletion region that results in peak splitting. Changes in the damping parameter are accounted for in all the four cases presented following the proportionality relation .

The spectrum variation becomes more complex when we consider the 3Layer case. Here, the two carrier populations in layers *L*_1_ and *L*_2_ can lead to a double peak in the absorption spectrum. We highlighted the main peak and the shoulder peak by green lines in the map. The position, intensity, and shape of the two peaks are then related to all the six aforementioned simulation parameters, and the effect of selective change of one of these leads to a nontrivial modification of the spectrum. Here, we report the most significant parameter variations, which will be useful to interpret the experimental data trends reported in the next sections. In particular, Figure 3a,b shows, respectively, the LSPR blueshift associated with increasing the carrier concentration *n*_*e*_*L*1__ and *n*_*e*_*L*2__ in each layer. The shoulder peak, represented by the green line at the higher wavenumber in both the spectral maps, disappears when the varying carrier concentrations reach comparable values. In particular, in Figure 3a, this happens when *n*_*e*_*L*1__∼*n*_*e*_*L*2__ = 5 · 10^20^ cm^–3^, whereas in Figure 3b, it is true under the condition *n*_*e*_*L*2__∼*n*_*e*_*L*1__ = 1 · 10^21^ cm^–3^. We remark that the simulations reported consider the damping factor variation associated with the carrier concentration change to be consistent with the physical modification associated to each region by the proportionality equations  and . It is crucial to include such dependency because the carrier concentration affects both directly and indirectly the LSPR peak evolution through γ. When we vary instead the geometrical composition of the NC by varying *L*_1_ while keeping DL constant (i.e., the active region and *F*_2_ constant), we observe a transition from double peaks to single peaks with increasing *L*_1_ (Figure 3c). On the contrary, if we increase *L*_2_ to the detriment of the depletion layer, we obtain the splitting of the peak and the increase in the main peak intensity (Figure 3d). Both geometrical modifications take into account the associated variation to the damping parameter as discussed above. Hence, the double peak appearance and peak splitting effects can be observed both when growing a shell around a core (geometrical change) and by varying the carrier density (via photodoping).

In the following sections, we will exploit the results obtained in these spectral plots, which are graphically intuitive to understand, to interpret the experimentally observed evolution of the peaks and parameters and relate them to physical changes of internal carrier density distribution, avoiding the possible overfitting of parameters.

### Photodoping of Uniform NC

**Figure 4:**
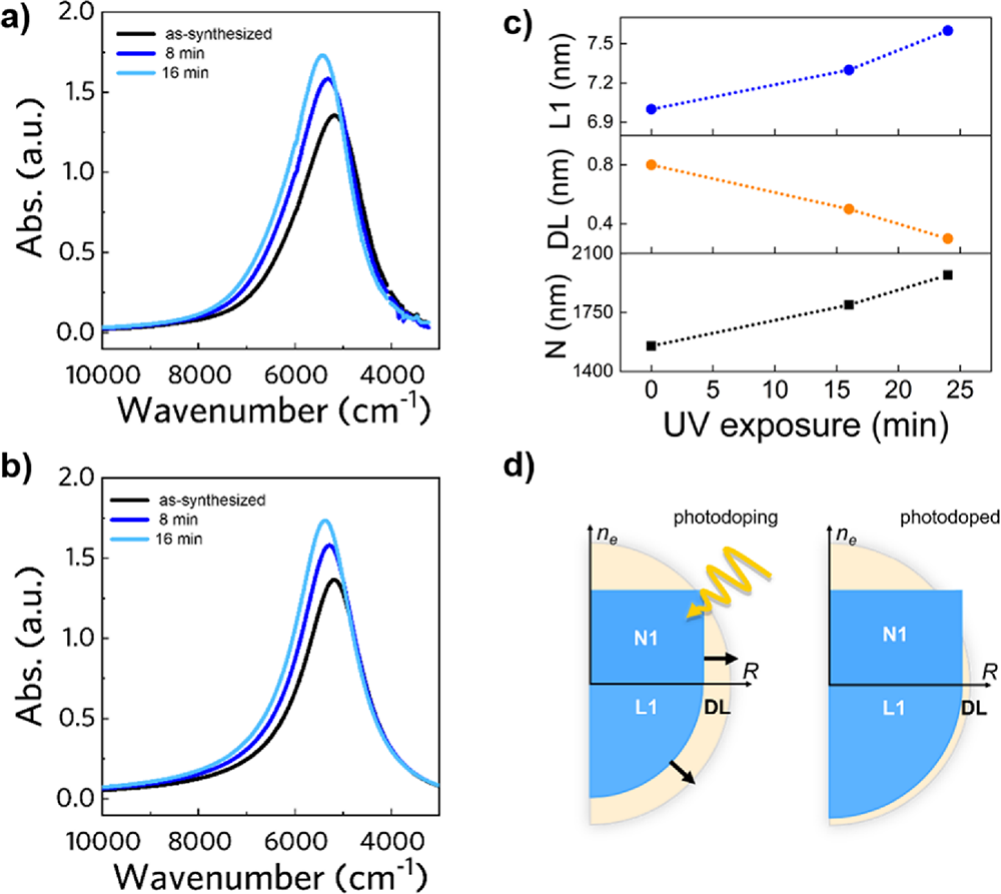
Photodoping of uniformly doped NC. (a) Evolution of the absorption spectrum upon photodoping and (b) corresponding simulations using the multilayer (two-layer) model. Experimental data from ref (9). (c) Evolution of the depletion layer (DL), active region (*L*_1_), and total number of free carriers (*N*) upon photodoping. (d) The approximated carrier profile structure of uniformly doped NC is depicted before and after the photodoping process. In the upper plot, the carrier concentration is reported for the as-synthesized and photodoped case; in the bottom part, a quarter of an NC section is shown instead. The colors are related to the two dielectric regions that approximate the NC: in blue, the core region; in yellow, the depletion region. Arrows represent the net effect of photodoping that leads to core region expansion to the detriment of the depletion layer. Panel a adapted from ref (9). Copyright 2022, Springer Nature. Copyright under the terms of the Creative Commons CC-BY license.

Irradiating NCs with photons that have an energy larger than the energy bandgap has the effect of generating *e*^–^/*h*^+^ pairs.^11,13,36^ In the photodoping process, the photogenerated carriers accumulate within the nanosystem and can affect the surface regions of the NC. Consequently, the total number of free carriers increases. From the model, as shown below, it becomes clear that this photocharging results effectively in the shrinking of the depletion layer. In the experimental case under investigation, we consider a solution of homogeneous ITO NCs with an average radius size *R*_NC_ = 7.8 nm and doped with 9.7% of Sn.^9^ The as-synthesized ITO NCs (black line in Figure 4a) show the typical LSPR peak in the NIR spectral range. After photodoping for several minutes, the spectra increase in intensity and blueshift (light and dark blue curves). Details about the experimental procedures are found in the original reference.^9^ In the following, we implement the above described 2L-model to reproduce these results. The NC is made of ITO, and it is necessary to take into account a depletion region as a result of the Fermi level pinning.^19^ Thus, the two-layer model is the most appropriate. The core fraction *L*_1_ is occupied with carriers (*N*_1_), and it is surrounded by the depleted region (DL). The solution concentration ρ_NC_ was determined experimentally by ICP-OES, whereas the average NC radius *R*_NC_ was measured by TEM imaging. These parameters are fixed during the fit.^9^

From the remaining three simulation parameters *F*, *n*_*e*_*L*1__, and γ_*L*1_, only *F* and *n*_*e*_*L*1__ are allowed to freely change, whereas the damping parameter γ_*L*1_ is constrained to follow the aforementioned proportionality relation  for all the simulations even though it is not fixed because the bulk mean free *l*_bulk_ path can vary according to its dependence with the carrier density. Notably, the experimental spectra are reproduced reasonably well by considering just an expansion of the core *L*_1_ (i.e., shrinking of the depletion layer) expressed by the variation of the geometrical factor *F*. In fact, the blueshift and the broadening of the LSPR peak experimentally observed can be associated with the reported trend shown in Figure 2c, where the core enlarges to the detriment of DL. Figure 4b shows the resulting simulation of the spectra. For a graphical detailed comparison of each spectra with the corresponding simulated one, we refer to the Supporting Information Figure S4. The derived parameters change is depicted in Figure 4c, where it is shown that the evolution of the spectra after photodoping is determined to be a result of both the introduction of the photogenerated carriers and the modification of the NC carrier profile. In fact, the total number of electrons *N* contributing to the LSPR increases, with the photogenerated carriers being stored in the expanded *L*_1_, which enlarges (i.e., *R*_*L*1_ increases) to the detriment of DL. No change in carrier density *n*_*e*_*L*1__ or in solution concentration ρ_NC_ is necessary. In fact, the Fermi level rise (i.e., increase in *n*_*e*_*L*1__) can be excluded because the associated blueshift would be too large compared to the experimental value and the damping parameter change would not be consistent with the experimental one.

In Figure 4d, we schematically sketched the carrier density profile as a step function versus the radius of the NC. The active, doped region expands after photodoping, and the depletion layer DL shrinks. The important role of the depletion layer in this type of nanocrystals has been first highlighted by Zandi et al.^19^ in the as-synthesized case and later used by Agrawal et al.^20^ to describe the tuning of ITO NCs upon electrochemical charging and in our recent paper to describe the active tuning of the same NCs upon photodoping.^9^ In fact, it is not possible to reproduce the spectral evolution of charge carrier injection in these nanocrystals if not implementing a two-layer model. In the following, we provide a discussion that highlights the physically doubtable results of a one-layer model for fitting the spectra. As a matter of fact, if we consider a uniform NC in the model, i.e., a one-layer model, it is possible to simulate simultaneously the experimental blueshift and the relative peak increase only by changing either both the carrier concentration *n*_*e*_*L*1__ and the damping parameter γ_*L*1_ or both the carrier concentration *n*_*e*_*L*1__ and the NC volumetric concentration (ρ_NC_). In the first case, the fit can be successfully performed when reducing the value of the damping parameter γ_*L*1_ while simultaneously increasing *n*_*e*_*L*1__. However, this result violates the relationship between the damping term and the carrier concentration, ,^33^ and is hence not reasonable. In the other case, both *n*_*e*_*L*1__ and ρ_NC_ increase, which are physically not reasonable because, as mentioned above, the concentration ρ_NC_ of the NCs must be preserved because the photodoped sample remains the same. Therefore, it is not possible to simulate the obtained evolution of the spectra with physically meaningful parameters considering just a uniform doped NC (one-layer model), and a two-layer model is necessary.

### Shell Growth

**Figure 5:**
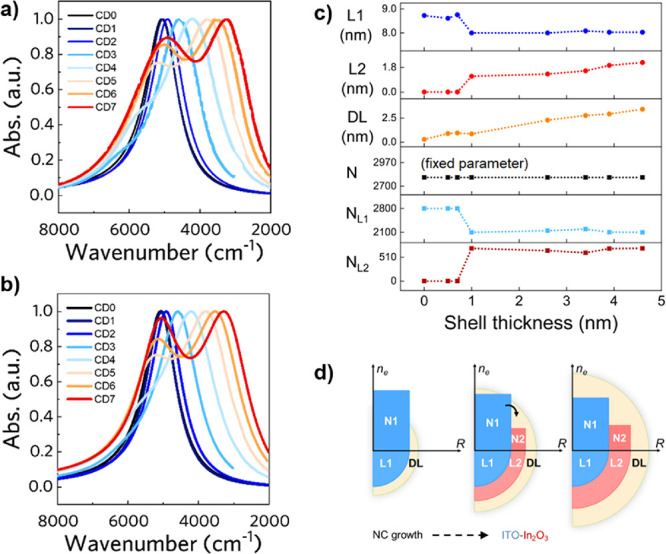
Growth of core–shell NCs simulated with the multilayer model. (a) Experimental absorption spectrum of ITO core NC with increasing shell thickness and (b) corresponding simulations using the multilayer model. Experimental data from ref (22). (c) Evolution of the main geometrical (DL, *L*_1_, *L*_2_) and electronic (*N*_*L*1_, *N*_*L*2_, *N*) parameters upon shell growth. (d) Scheme illustrating the procedure followed to replicate the experimentally observed trend. A spillover of electrons from the core into the shell is simulated by the three-layer model. The second layer (*L*_2_), which expands upon shell growth, is responsible for the appearance of the second plasmonic mode. Panel a adapted from ref (22). Copyright 2020, American Chemical Society.

In the next case, we consider the experimental spectra corresponding to doped-core NCs of ITO on which a variable-thickness shell of undoped In_2_O_3_ was grown. The model system of ITO-IO NCs has been published previously by Gibbs et al.^16^ The core is represented by ITO doped with 5% Sn and has a total radius *R*_NC_ of 9.0 nm. The shell thickness varies from core-only (sample CD0) to a maximum of 4.7 nm (CD7). Further details on the NC synthesis, characterization, and spectra acquisition are found in the original publication.^22^ In Figure 5a, the spectra are shown with an increasing shell thickness from black to red. It is notable that, upon shell growth, the main LSPR peak redshifts. This is a result of the increased surrounding dielectric constant.^37^ Moreover, when a shell layer reaches a thickness comparable to the depletion layer width of the core-only case (1–2 nm^19^), a shoulder in the absorption spectra appears that evolves to a double peak with increasing shell thickness.^22^ The observed spectrum evolution can be associated with a shell-growth-like behavior, similar to the one reported in the simulations of Figure 3d. Nevertheless, here, the total radius *R*_NC_ changes as well, and its effect must be taken into account in the following simulation discussion.

To simulate the experimental spectra, each sample is associated with its relative solution concentration ρ_NC_ measured, as mentioned above, by ICP analysis (see ref (22) for details). For all the samples, the average NC radius *R*_NC_ is measured by TEM imaging and considered a fixed parameter. Before describing the parameter evolution upon core–shell growth in detail, we present a range of initial assumptions that arise by carefully analyzing the core-only sample (CD0). We make the crucial hypothesis that the total number of free carriers (*N*) contributing to the LSPR in all samples remains constant upon shell growth. This assumption is reasonable, as no additional Sn dopants are added because the number of free electrons associated with the In_2_O_3_ shell layer is negligible^36,38^ and the surface dopant activation would result in a blueshift of the peak for the smaller shell grown, but after the activation of dopants, *N* would remain constant.^8^ To reproduce a step-function-like carrier density profile observed previously by us,^9^ we make the assumption that the number of carriers locally present in the core (*N*_*L*1_) and shell (*N*_*L*2_) can vary, whereas the total number of carriers (*N* = *N*_*L*1_ + *N*_*L*2_) is fixed for all the samples. These values are introduced into the three-layer model. For the cases where the In_2_O_3_ shell layer is smaller than the core-only DL (<1 nm, samples CD0–2), we consider a two-layer model (*L*_2_ = 0). For all the other samples where a second mode appears (samples CD3–7), we consider the three-layer model (i.e., *L*_2_ > 0). In fact, the presence of a double peak in samples CD3–7 is a signature that a further region contributing to the plasmon resonance exists.

Determining *N* for sample CD0 is critical as it affects the subsequent spectrum evolution upon shell growth. Further, *N* uniquely defines the simulation parameters *F* and *n*_*e*_*L*1__. The maximum value of *N* can be determined by fitting the CD0 sample and assuming the absence of a depletion layer (DL = 0 nm, *R*_*L*1_ = *R*_NC_), as adding a nonzero DL will ultimately result in a smaller value of *N*. Second, we determine the minimum value of *N* by assuming that the DL in CD0 does not exceed 1 nm.^22^ This results into a range of values for *N* (2290 < *N* < 3010). Eventually, for each guess of *N* in this range, a different set of parameters can be determined for all the CD1–7 spectra. If the initial *N* is not adequate, the simulation of the following spectrum represents either some unphysical simulation parameters evolution (γ_*L*1_ in particular) or the impossibility of reproducing accurately the spectrum. Therefore, we additionally fix the damping parameter γ_*L*1_ determined in the CD0 case, recalling the dependence of .^33^ In the following, we present our best results for *N* = 2500. We remark here that reasonable fits can also be obtained for other values of *N* with slight deviations in the fit values (see another example in the Supporting Information). The simulated spectra obtained by fixing *N* = 2500 are reported in Figure 5b, whereas a one-by-one comparison of each spectrum with the corresponding simulated one can be found in Figure S5 in the Supporting information. The parameter variation is depicted in Figure 5c. In particular, the total number of electrons in the core *N*_*L*1_ diminishes upon shell growth. The difference (*N* – *N*_*L*1_) represents the electrons stored in the shell region (*N*_*L*2_), which increases together with the shell. The core radius *R*_*L*1_ continuously decreases, whereas both the shell region *R*_*L*2_ and the depletion layer DL expand upon shell growth. Eventually, the damping parameters γ_*L*1_ and γ_*L*2_ are constrained by *l*_bulk_ range and are free to evolve with shell growth following the respective proportionality relations^33^ and .

Figure 5d depicts a scheme of the evolution of the carrier density profile and geometrical depletion layer variation upon shell growth for samples CD0, CD4, and CD7. We can observe that the NC growth is associated with the redistribution of electrons in the shell and with an expansion of the DL. The carrier density profile that reproduces the observed double peak evolution can be well approximated with a step-like function, accounting for the DL and the carrier redistribution between *L*_1_ and *L*_2_. The implementation of the 3L-model allowed the extraction of important information on the local electronic structure of core–shell ITO-IO NCs, including the depletion layer evolution avoiding unphysical change in parameters (such as damping parameters or the total number of electrons *N*).

### Photodoping of Core–Shell NCs

**Figure 6:**
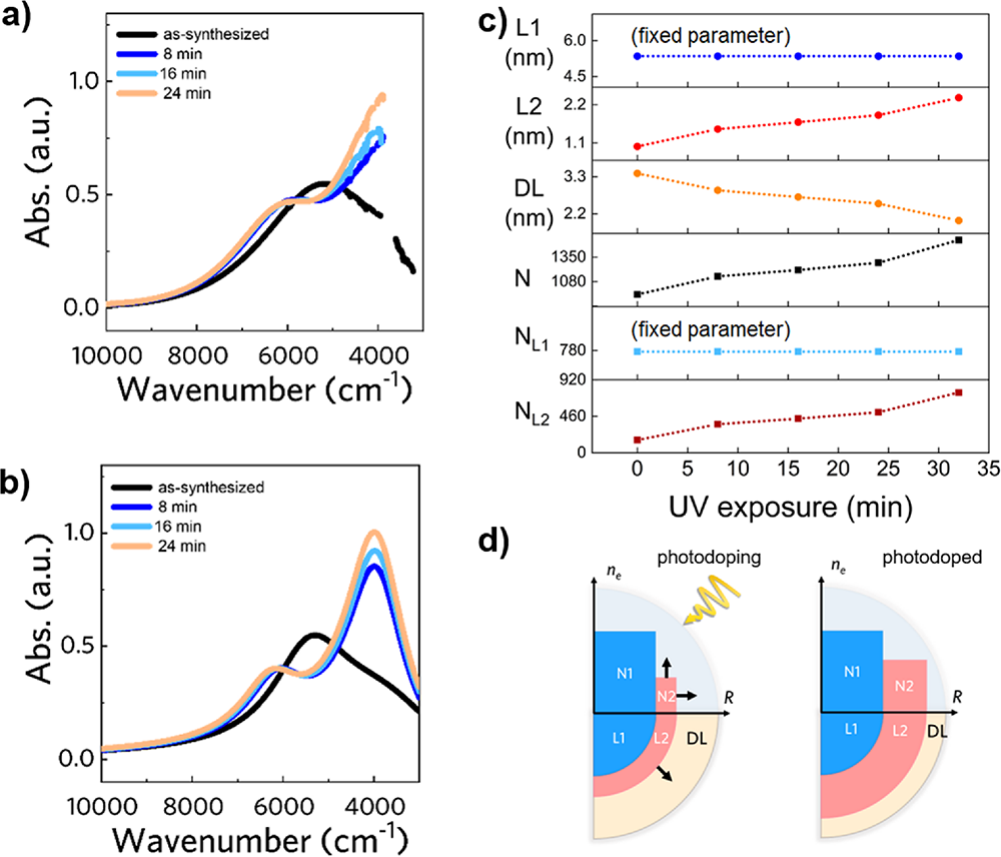
Photodoping of ITO NCs and corresponding simulation with the multilayer (three-layer) optical model. (a) Evolution of the absorption spectrum of ITO-In_2_O_3_ core–shell NCs under UV exposure shows plasmon peak splitting. Experimental data from ref (9). (b) The corresponding simulated spectra can be obtained with the three-layer model, considering a core–shell depletion structure. (c) Evolution of the main geometrical (DL, *L*_1_, *L*_2_) and electronic (N1, N2, N) parameters upon shell growth. (d) Scheme illustrating the procedure followed to replicate the experimentally observed trend. Upon photodoping, the second layer (*L*_2_) expands and increases its concentration (N2). For peak splitting, the NCs undergo a two-step process: initially, photodoping causes plasmonic splitting by increasing the contribution of charges in the second layer, which is modeled by a decrease in damping parameter γ_*s*_; after that, the addition of extra photoelectrons causes the peaks to blueshift and increase in intensity. Panel a adapted from ref (9). Copyright 2022, Springer Nature. Copyright under the terms of the Creative Commons CC-BY license.

As the last case, we analyzed the effect of post-synthetic modification (via photodoping) on core–shell NCs. Specifically, by photodoping ITO-IO core–shell NCs, a light-induced peak splitting of the plasmon resonance was experimentally observed (see Figure 6a). The as-synthesized NCs are composed by a Sn-doped core (10.8%) of ITO surrounded by a 4.25 nm shell layer. The average radius *R*_NC_ = 9.75 nm is measured with TEM, and the solution concentration ρ_NC_ = 3.0 · 10^13^ mL^–1^ is determined by ICP-OES. Details about the synthesis and experimental procedures are reported in the original reference.^9^ The as-synthetized case (black curve) shows a broad plasmon peak, which is associated with the main core plasmon contribution and a damped thin shell layer. After photodoping (blue to orange curves), the spectra show a peak splitting leading to double peak resonance. From the titration of the photodoped solution, we acknowledge that electrons have been stored in the NCs, which justify the increase in peak intensity but not the splitting of the peak. The observed evolution can be explained considering the simulated case reported in Figure 3d, where the shell expansion at the expense of the depletion region and the increase in the number of carriers present in the shell determine a trend comparable to the experimental one .Here, a three-layer model is used to simulate the spectra, accounting for the core, shell, and DL. The corresponding simulated spectra are shown in Figure 6b, whereas a one-by-one comparison is reported in Figure S6 in the Supporting Information. The derived parameters are reported in Figure 6c. Among the simulation parameters used, the core parameters, i.e., core carrier concentration *n*_*eL*1_ and damping γ_*L*1_, are kept constant because the core region *L*_1_ is not altered by the photodoping process.^9^ Photodoping deeply affects the surface region, modifying the shell (*R*_*L*2_) and depletion layer (DL). The depletion layer DL shrinks with increasing photodoping time, whereas *R*_*L*2_ increases. We found that a consistent decrease in the damping parameter γ_*L*2_ is the fundamental key to reproducing the peak splitting. This reduction of γ_*L*2_ is supported by the inverse proportionality to *R*_*L*2_(33), which is correlated to the shell expansion. The carriers photogenerated with photodoping are stored in the shell layer (*N*_*L*2_ increases), contributing to the peak intensity increase and to the blueshift of the spectrum with photodoping time (Figure 6d). Although with a two-layer model it is possible to reproduce the observed spectrum evolution by applying the same consistent reduction of the shell damping parameter γ_*L*2_, this change is not justified physically as the shell expansion is absent, and γ_*L*2_ should increase with increasing *n*_*e*_*L*2__. This highlights that the three-layer model is also required in this latter scenario to reproduce the experimental spectrum with a physically meaningful parameter evolution.

## Conclusions

In this work, we investigated the application of multilayer optical models for studying the evolution of optical spectra of MO NCs. We first provided a systematic study of two-layer and three-layer models, used respectively as reference for core and core/shell structures, both surrounded by a depletion layer. We simulated the optical spectra taking into account the presence of the depletion layer at the surface and varying selectively one parameter. Differently from previous studies, in the simulations, we took into account the interdependence among the parameters, with particular emphasis on the damping factor γ_*Lx*_ dependence with electron concentration *n_e_Lx__* and layer size *R_Lx_*. In the case of the two-layer core/DL structure, the LSPR peak evolution is easily interpretable, whereas in the case of a core/shell structure surrounded by a depleted layer, we obtained nontrivial evolution of the two peaks present in the LSPR. In particular, we showed that peak splitting can derive from expansion of the shell layer into the depletion layer or to the detriment of the core. Then, we exploited these simulation results to qualitatively interpret the spectrum evolution of three experimental study cases that have been recently published in the literature. We employed such qualitative comparison between simulations and experimental trend to choose the correct model to employ for fitting the experimental data and to obtain a physical evolution of simulation parameters. We showed that for all three cases, it is crucial to take into account the depletion layer and it is fundamental to make a hypothesis on parameter evolution to avoid multiple solution results. In the case of photodoping for both core and core/shell NCs, the experimental LSPR evolution can be explained with the expansion of the active region in the depletion layer. Conversely, for the growth of a shell on a core structure, the double peak appearance derives from an internal rearrangement of the electronic band profile due to the appearance of a shell region that expands with nanoparticle growth. We foresee that this simulation approach to the multilayer model can be used both as a powerful tool for getting insight into the internal carrier density profile and to engineer NC optoelectronic properties as well.^16,20^ We highlight that these models are extendable to other plasmonic nanocrystals with core–shell architectures and that this work will contribute to a more complete understanding of the proper implementation of such models in the community.

## Supporting Information Available

The data underlying this study are openly available in Zenodo at https://doi.org/10.5281/zenodo.7515557. The Supporting Information is available free of charge at https://pubs.acs.org/doi/10.1021/acs.jpcc.2c05582.

Additional theoretical calculations on absorption vs scattering coefficients and relative plots; tables reporting the simulation values employed and the fitting parameters; additional figures reporting discrete simulation spectra for different parameter variations for the two-layer and three-layer optical model; figures reporting simulated and experimental spectrum detailed comparison; and discussion on simulation details of the shell-growth case (PDF)
