# Supplementary material for: Optical Modeling of Plasmonic Nanoparticles with Electronically Depleted Layers
Source: J Phys Chem C Nanomater Interfaces. 2023 Jan 11;127(3):1576–87. doi: 10.1021/acs.jpcc.2c05582 (PMC9884077; doi:10.1021/acs.jpcc.2c05582)
Supplement: Supplementary file 1 — jp2c05582_si_001.pdf [file jp2c05582_si_001.pdf]

Supplementary Information

Optical Modeling of Plasmonic Nanoparticles with Electronically Depleted Layers

Nicolò Petrini[1,2,#], Michele Ghini[1,#], Nicola Curreli[1], Ilka Kriegel[1,*]

1 - Functional Nanosystems, Istituto Italiano di Tecnologia (IIT), via Morego 30, 16163 Genova, Italy
2 - Dipartimento di Fisica, Università degli Studi di Genova, Via Dodecaneso 33, 16146, Genova, Italy

[*]Email: ilka.kriegel@iit.it
[#]Equal contribution

Absorption vs Scattering

We discuss here the absorption and scattering contributions to the optical response of plasmonic nanocrystals. In particular, we examine the hypothesis that the scattering contribution is negligible for the NCs considered in this work by analyzing the analytical solution of Maxwell equations in the case of electromagnetic field interacting with spherical particle. The theoretical equations were firstly derived by Mie and lead to obtain the extinction (C_{ext}), scattering (C_{sca}), and absorption (C_{abs}) cross sections as[1,2] :

$$C_{ext} = \frac{2\pi}{k^2} \sum_{n=1}^{\infty} (2n+1) \mathrm{Re}\{a_n + b_n\} \tag{1}$$

$$C_{sca} = \frac{2\pi}{k^2} \sum_{n=1}^{\infty} (2n+1)(|a_n|^2 + |b_n|^2)$$

$$C_{abs} = C_{ext} - C_{sca}$$

Where k is the wavenumber of the impinging light calculated in the surrounding medium, n is the Bessel-function order. Then, the calculation of the wavelength-dependent complex scattering coefficients a_n and b_n becomes the focus. These coefficients depend on the size of the particle, its dielectric function, and the dielectric function of the surrounding medium. The scattering coefficient equations are:

$$a_n = \frac{m\psi_n(mx)\psi_n'(x) - \psi_n(x)\psi_n'(mx)}{m\psi_n(mx)\xi_n'(x) - \xi_n(x)\psi_n'(mx)} \tag{2}$$

$$b_n = \frac{\psi_n(mx)\psi_n'(x) - m\psi_n(x)\psi_n'(mx)}{\psi_n(mx)\xi_n'(x) - m\xi_n(x)\psi_n'(mx)}$$

Where x is the size parameter, defined as $k \cdot R$, and m is the relative refractive index, defined as $m = \sqrt{\frac{\varepsilon_{ITO}}{\varepsilon_m}}$, ψ_n and ξ_n are the Riccati-Bessel functions, accounting for the spherical symmetry of the problem.

To verify if the scattering contribution to the total extinction is negligible for a defined particle dimension, we evaluate the ratio between the scattering and absorption coefficients C_{sca} and C_{abs} for nanoparticles radii R varying from 1 nm to 500 nm. We consider the wavelength dependent response between 100 nm and 10 µm wavelengths, considering the refractive index of uniform Metal Oxide (in this case ITO) nanocrystals with typical free-carrier concentration of 10^{21} cm^{-3}. In **Figure S1a** we reported the ratio results for each wavelength (y axis), varying the NC radius (x-axis). The red contour line defines where $C_{sca} = C_{abs}$. For each wavelength and radius for which is verified $C_{sca} << C_{abs}$ (hence the ratio is high), the scattering part is negligible and the hypothesis holds. If we plot the ratio for the wavelength in which the absorption has its maximum, we obtain the plot in **Figure S1b**. From the reported trend, we can more easily state that for the NC dimension considered in this article (9 nm < R < 20 nm), scattering is negligible and hence we can approximate the absorption with extinction.

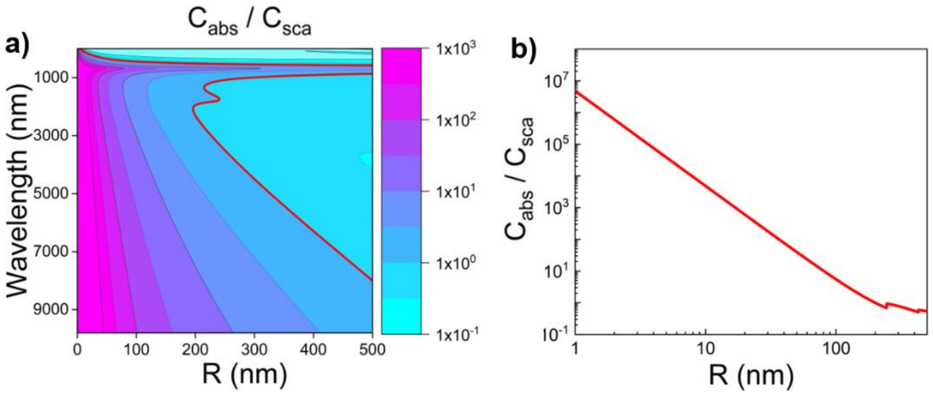

Figure S1 – Scattering and absorption contributions. a) Ratio between the absorption (C_{abs}) and the scattering (C_{sca}) cross sections for various wavelengths (y axis) and NC radii (x-axis). The red contour line defines where the two cross sections are equal. **b**) Plot of the same ratio for the wavelength in which the absorption has its maximum. From the reported trend, we can conclude that for the NC dimension considered in this article (9 nm < R < 20 nm), scattering is negligible and hence we can approximate the absorption with extinction.

Parameters simulations for 2-Layer and 3-Layer models

Here we report the default simulation parameters used for 2-Layer and 3-Layer simulations for **Figure 2** and **Figure 3** in the main text. Table S1 reports the simulation parameters and derived parameters for 2-Layer case while Table S2 for the 3-Layer case.

Table S1

Default parameter	Value
R_{NC}	10 nm
ρ_0	$6.7 \cdot 10^{12}$ mL^{-1}
$n_{e_{L1}}$	$1.0 \cdot 10^{21}$ cm^{-3}
R_{L1}	7 nm
DL	3 nm

F	0.343
γ_{L1}	1500 cm^{-1}

Table S2

**Default parameter**	**Value**
R_{NC}	10 nm
ρ_0	$2.182 \cdot 10^{13}$ mL^{-1}
$n_{e_{L1}}$	$1.0 \cdot 10^{21}$ cm^{-3}
$n_{e_{L2}}$	$5.0 \cdot 10^{20}$ cm^{-3}
R_{L1}	6 nm
R_{L2}	2 nm
DL	2 nm
F_1	0.422
F_2	0.512
γ_{L1}	1000 cm^{-1}
γ_{L2}	1737 cm^{-1}

Table S3

	**As-synthesized**	**8 min**	**16 min**
$n_{e_{L1}}$(cm^{-3})	$1.08 \cdot 10^{21}$	$1.08 \cdot 10^{21}$	$1.09 \cdot 10^{21}$
F_1	0.70906	0.82083	0.89322
γ_{L1} (cm^{-1})	1481	1450	1429
ρ_0 (mL^{-1})	$2.67 \cdot 10^{13}$	$2.67 \cdot 10^{13}$	$2.67 \cdot 10^{13}$
N	1551	1795	1972
R_{L1}(nm)	7	7.3	7.6
DL (nm)	0.8	0.5	0.3

Table S4

	**CD0**	**CD1**	**CD2**	**CD3**	**CD4**	**CD5**	**CD6**	**CD7**
$n_{e_{L1}}$(cm^{-3})	$1.05 \cdot 10^{21}$	$1.08 \cdot 10^{21}$	$1.03 \cdot 10^{21}$	$9.21 \cdot 10^{20}$	$1.01 \cdot 10^{21}$	$1.03 \cdot 10^{21}$	$9.94 \cdot 10^{20}$	$9.96 \cdot 10^{20}$
$n_{e_{L2}}$(cm^{-3})	0	0	$1.55 \cdot 10^{19}$	$5.64 \cdot 10^{20}$	$5.64 \cdot 10^{20}$	$4.61 \cdot 10^{20}$	$3.81 \cdot 10^{20}$	$3.57 \cdot 10^{20}$
F_1	1	1	0.96384	0.86775	0.57821	0.45238	0.44236	0.35072
F_2	0.78239	0.64325	0.66013	0.68303	0.46598	0.43538	0.42652	0.40828
γ_{L1} (cm^{-1})	1120	1048	1126	1107	1139	1011	1043	1005
γ_{L2} (cm^{-1})	100000	100000	2895	2739	2357	2628	2333	2691
ρ_0 (mL^{-1})	1	1	1	1	1	1	1	1
N	2500	2500	2500	2500	2500	2500	2500	2500
N_{L1}	2500	2500	2499	2287	1775	1622	1686	1503
N_{L2}	0	0	1	213	725	878	814	997
R_{L1}(nm)	8.2931	8.2007	8.3429	8.4	7.4922	7.2146	7.3988	7.115
R_{L2}(nm)	0	0	0.10305	0.40672	1.501	2.1836	2.3116	2.9742
DL (nm)	0.7069	1.2993	1.25405	1.19328	2.6068	3.0018	3.1896	3.5108

Table S5

	**As-synthesized**	**8 min**	**16 min**	**24 min**
$n_{e_{L1}}$(cm^{-3})	$1.21 \cdot 10^{21}$	$1.21 \cdot 10^{21}$	$1.21 \cdot 10^{21}$	$1.21 \cdot 10^{21}$
$n_{e_{L2}}$(cm^{-3})	$3.73 \cdot 10^{20}$	$5.12 \cdot 10^{20}$	$5.21 \cdot 10^{20}$	$5.35 \cdot 10^{20}$
F_1	0.59805	0.47642	0.43701	0.40183
F_2	0.27625	0.34678	0.37805	0.41115
γ_{L1} (cm^{-1})	1512	1512	1512	1512
γ_{L2} (cm^{-1})	3508	1661	1637	1629
ρ_0 (mL^{-1})	$3.02 \cdot 10^{13}$	$3.02 \cdot 10^{13}$	$3.02 \cdot 10^{13}$	$3.02 \cdot 10^{13}$
N	939	1138	1208	1288
N_{L1}	778	778	778	778
N_{L2}	161	360	430	510
R_{L1}(nm)	5.35	5.35	5.35	5.35
R_{L2}(nm)	1	1.5	1.7	1.9
DL (nm)	3.4	2.9	2.7	2.5

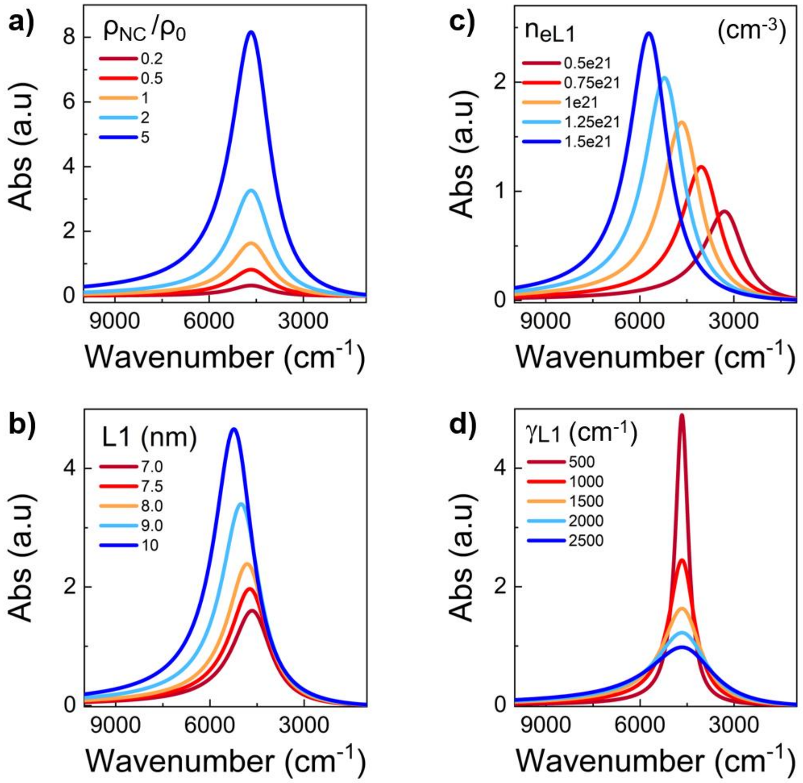

Figure S2 –Discrete simulation evolution of the absorption spectrum of homogeneous ITO NCs as a function of several parameters of the Multi-Layer (2-Layer) optical model. a) Increasing the concentration of the NC solution ρ_{NC} with respect to a base concentration $\rho_0 = 6.7 \cdot 10^{12}$ mL^{-1} increases linearly the absorption intensity maximum of the LSPR. An increase of the carrier density level $n_{e_{L1}}$(**b**) or of the layer size L_1 (**c**) significantly affects the peak lineshape, blueshifting the peak position and increasing its intensity. We remark that the damping parameter changes accordingly to the relation $\gamma_{L1} \propto \sqrt[3]{n_{e_{L1}}} \cdot R_{L1}^{-1}$ **d**) Changes in the damping parameter affect both the width of the LSPR and its intensity, but not the peak frequency position.

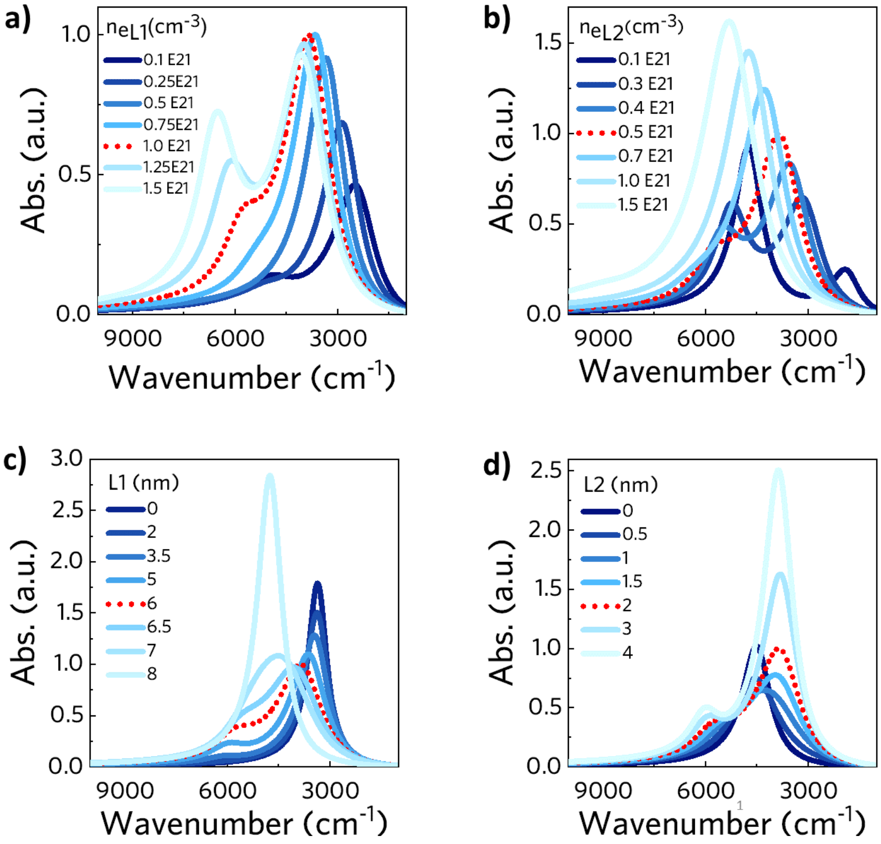

Figure S3 – Discrete simulation evolution of the absorption spectrum of core/shell ITO/IO NCs as a function of several parameters of the Multi-Layer (3-Layer) optical model. a) and (**b**) show respectively the variation of core carrier density level $n_{e_{L1}}$ and shell $n_{e_{L2}}$. (**c**) and (**d**) depict instead the geometrical variation respectively of L_1 to the detriment of shell, and L_2 to the detriment of DL For all four simulations the damping parameter changes accordingly to : $\gamma_{L1} \propto \sqrt[3]{n_{e_{L1}}} \cdot R_{L1}^{-1}$ and $\gamma_{L2} \propto \sqrt[3]{n_{e_{L2}}} \cdot R_{L2}^{-1}$

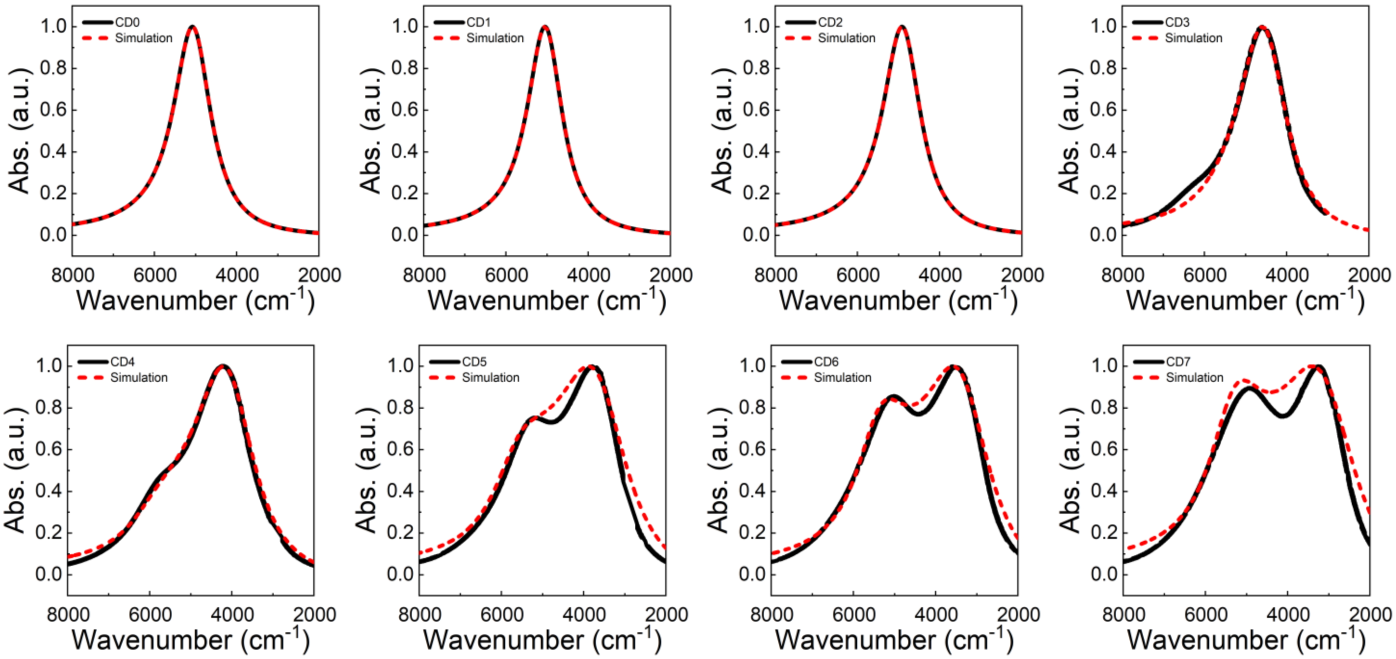

Figure S5 – Shell growth. In black it is reported the experimental spectrum simulation, in red the corresponding simulation.

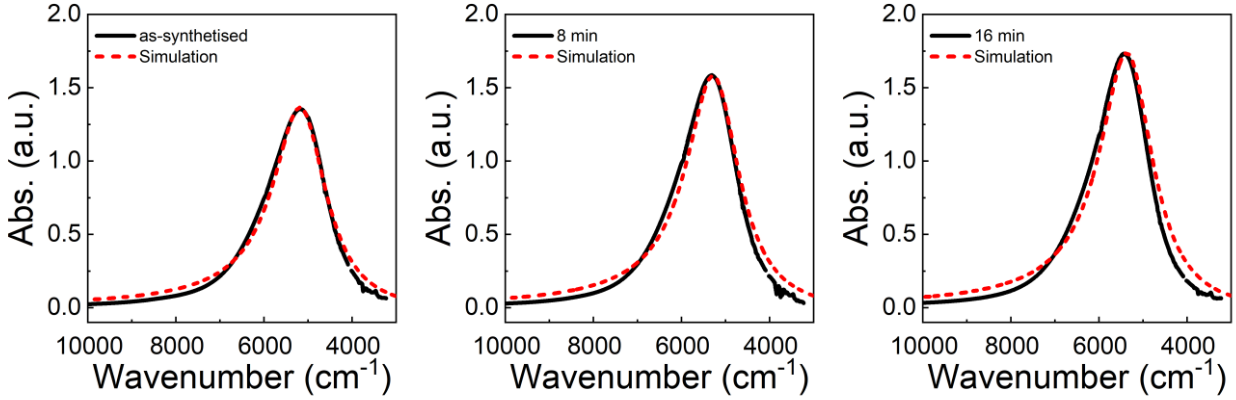

Figure S4 – Photodoping of Uniform Core. In black it is reported the experimental spectrum simulation, in red the corresponding simulation.

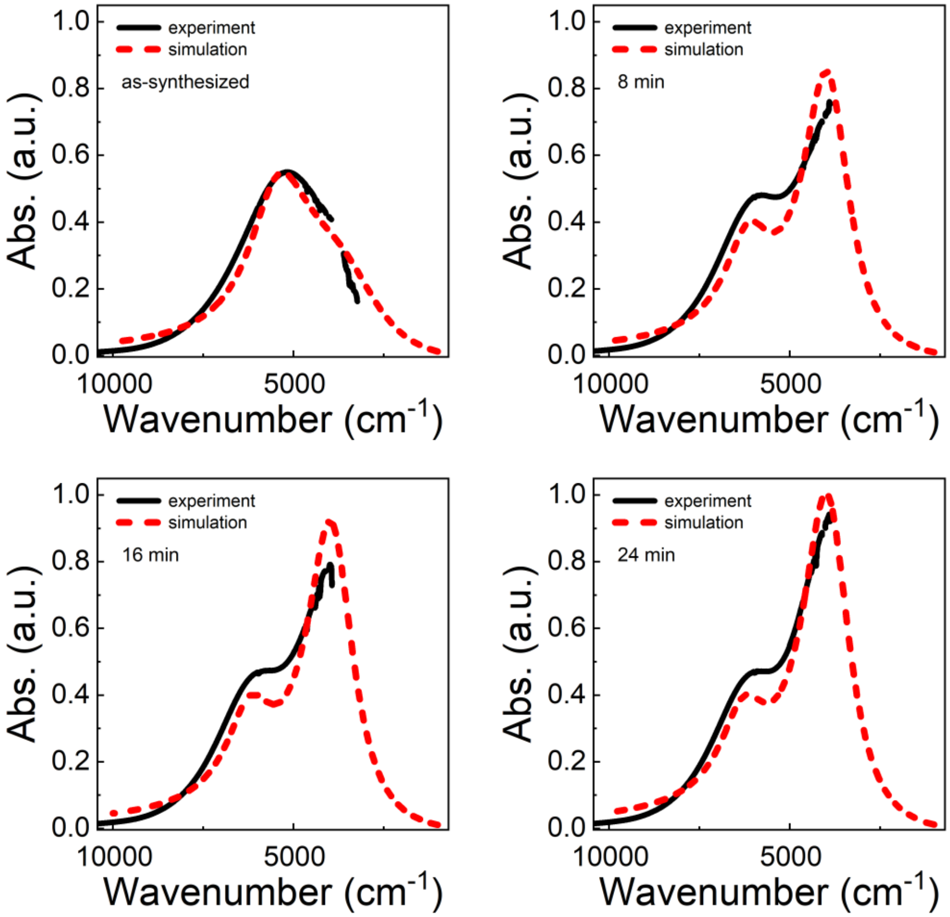

Figure S6 – Photodoping of core-shell NCs. In black it is reported the experimental spectrum simulation, in red the corresponding simulation.

Discussion on fixing total electron number N

In the experimental spectra reported in the "Shell growth" section, the appearance of the second peak leads to consider a second metallic layer (L_2) in the optical model[3]. Moreover, the depletion layer still needs to be considered at the surface. This leads to implement a 3-Layer model, with associated a set of 6 free parameters which can lead to multiple solution for reproducing the same spectrum. To avoid overfitting, some physical constraints on the parameters needs to be added, and in particular it is physically reasonable to consider as fixed the total number of free electrons N present in the nanocrystals, since the growth of In_2O_3 shell layer does not introduce a significant number of free carriers.[4] The range of possible N can be determined considering the spectrum of CD0, for which 2-Layer model is sufficient, and considering the possible combinations of F and $n_{e_{L1}}$. In fact, for each combination of F and $n_{e_{L1}}$ we can calculate the total number of electrons N. The maximum possible value for N is determined by the conditions of uniform core with DL=0 nm and $R_{L1} = R_{NC}$, leading to a maximum of $N = 3010$ carriers. Physically, we can moreover

reduce the possible combination of F and $n_{e_{L1}}$, considering that the depletion layer for the uniformly-doped NC considered will not exceed 1 nm thickness[5], leading to $2290 < N < 3010$. Eventually, for each guess of N in this range, a different set of parameters can be determined for all the CD0-7 spectra. Here in **Figure S2** we report the simulation spectra obtained with N=2500 and N=2800 and the relative parameter variation. Comparing the spectra and the parameter evolution, we can highlight that while in the case of N=2800 the CD3 shoulder is better reproduced, nevertheless the parameter evolution presents an abrupt jump when the shoulder peak appears, i.e. in the CD3 case. We highlight that the estimation of the free electrons N can be refined and the real value might lie somewhere between 2500 and 2800. In order to calculate it more precisely, it would be necessary to determine it with further experiments, for example by using photodoping of sample CD0. In fact, considering the spectra evolution of only-core sample as reported in the "Photodoping of uniform NC" section, it is possible to uniquely determine the parameter set and thus free electron number N.

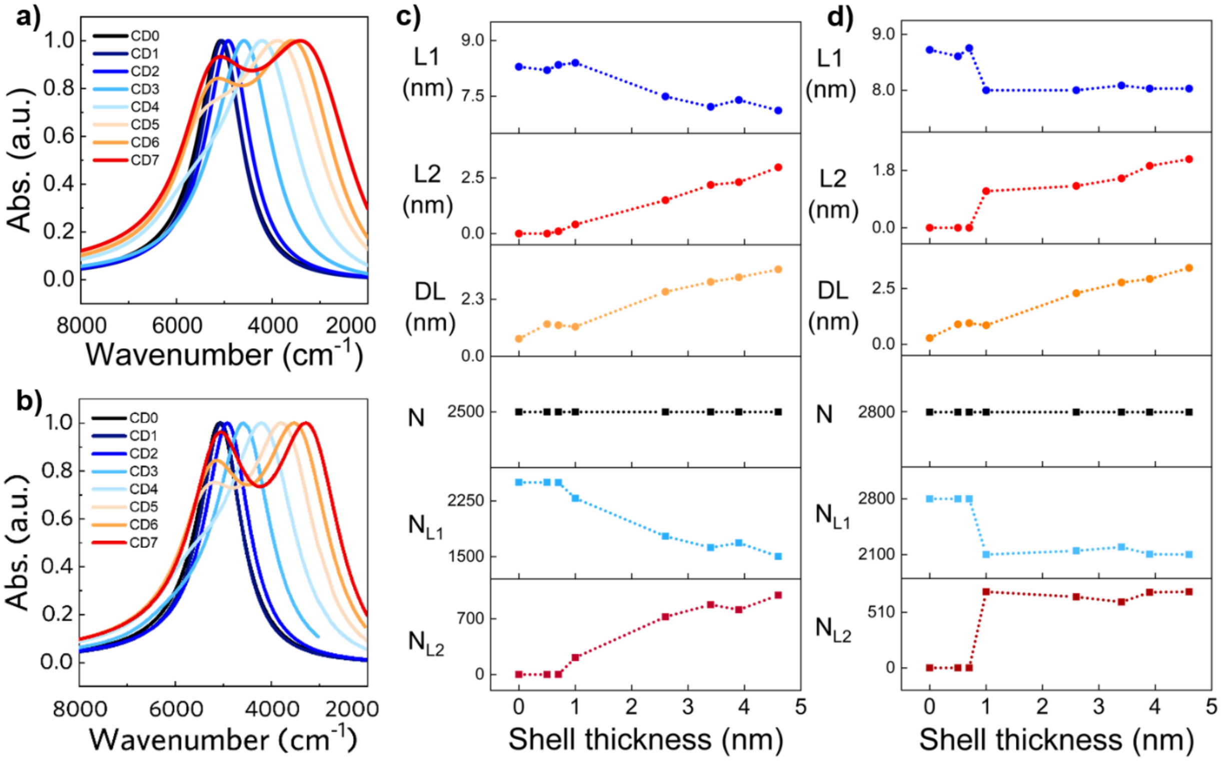

Figure S7 – Spectral evolution upon shell growth in core-shell NCs with fixed amount of electrons. Simulations of the optical response in the case of **a)** 2500 total electrons and **b)** 2800 total electrons fixed. Evolution of the main geometrical (DL, L_1, L_2) and electronic (N_{L1}, N_{L2}, N) parameters upon shell growth in the in the two cases of **c)** 2500 total electrons and **d)** 2800 total electrons.

REFERENCES

(1) Bohren, C. F.; Huffman, D. R. *Absorption and Scattering of Light by Small Particles*; Wiley, 1998. DOI: 10.1002/9783527618156.

(2) Matsui, H. Surface Plasmons in Oxide Semiconductor Nanoparticles: Effect of Size and Carrier Density. In *Nanocrystalline Materials*; Movahedi, B., Ed.; IntechOpen: Rijeka, 2019; pp 1–17. DOI: 10.5772/intechopen.86999.

(3) Gibbs, S. L.; Dean, C.; Saad, J.; Tandon, B.; Staller, C. M.; Agrawal, A.; Milliron, D. J. Dual-Mode Infrared Absorption by Segregating Dopants within Plasmonic Semiconductor Nanocrystals. *Nano Lett.* **2020**, *20* (10), 7498–7505. DOI: 10.1021/acs.nanolett.0c02992.

(4) Schimpf, A. M.; Lounis, S. D.; Runnerstrom, E. L.; Milliron, D. J.; Gamelin, D. R. Redox Chemistries and Plasmon Energies of Photodoped In2O3 and Sn-Doped In2O3 (ITO) Nanocrystals. *J. Am. Chem. Soc.* **2015**, *137* (1), 518–524. DOI: 10.1021/ja5116953.

(5) Zandi, O.; Agrawal, A.; Shearer, A. B.; Reimnitz, L. C.; Dahlman, C. J.; Staller, C. M.; Milliron, D. J. Impacts of Surface Depletion on the Plasmonic Properties of Doped Semiconductor Nanocrystals. *Nat. Mater.* **2018**, *17* (8), 710–717. DOI: 10.1038/s41563-018-0130-5.
